# PLT antigen discrepancy pattern among couples with recurrent abortion

**DOI:** 10.3389/fmed.2024.1291779

**Published:** 2024-04-08

**Authors:** Ghazal Ahmadzadeh Shad, Ali Ghasemi, Maryam Zadsar, Mojgan Shaeigan, Shahram Samiee, Ahad Zare

**Affiliations:** ^1^Iranian Blood Transfusion Organization Research Center, Tehran, Iran; ^2^Department of Biochemistry and Hematology, Faculty of Medicine Semnan University of Medical Sciences, Semnan, Iran; ^3^Infectious Disease Specialist, Blood Transfusion Research Center/High Institute for Research & Education in Transfusion Medicine/Microbiology Department, Tehran, Iran; ^4^Immunology, Blood Transfusion Research Center /High Institute for Research & Education in Transfusion Medicine, Immunohematology Department, Tehran, Iran; ^5^Biochemistry, Iranian Blood Transfusion Organization Research Center, Tehran, Iran; ^6^Sarem Fertility and Infertility Research Center, Sarem Cell Research Center, Sarem Hospital, Tehran, Iran

**Keywords:** antigens, human platelet, recurrent abortion, pregnancy, gynecology

## Abstract

**Background:**

Recurrent abortion refers to a condition of two or more consecutive pregnancies without known etiology affected by miscarriage before the completion of the 20th week of gestational age. However, several hypotheses have been proposed, but not much data are available concerning the relationship between human platelet antigens (HPAs) polymorphisms and recurrent abortion. This study was conducted to evaluate the genetic differences between HPA-1, −2, −3, −5, and − 15 in Iranian couples with a history of recurrent abortion.

**Methods:**

In this cross-sectional study, a total of 74 couples with at least 2 recurrent abortions without any known specified reasons enrolled in the study. HPA polymorphisms genotyping was performed by single-specific primer PCR. Genotype frequency was calculated using the Hardy–Weinberg equation.

**Results:**

A total of 39 couples (52.7%) had HPA genotyping partial mismatches. The most common partial mismatch pairs were found concomitantly on both HPA-15a and HPA-15b in three couples (4%), followed by two (2.7%) on HPA-3a and one (1.3%) in each HPA-2b and HPA-5b. There was a deviation from the Hardy–Weinberg equilibrium in the HPA-2 and -5 systems.

**Conclusion:**

The present study declared that partial mismatches of HPA-3 and -15 genotypes were common among Iranian couples due to the history of recurrent abortion and approximately half of the couples carried at least one HPA gene that was absent in their partners. Further studies might be helpful to clarify the association between HPA polymorphisms and recurrent abortion, such as an investigation into the alloantibodies against HPAs.

## 1 Introduction

Recurrent abortion is one of the major concerns in the field of gynecology, which causes a huge amount of distress and economic burden. It is defined as two consecutive spontaneous miscarriages before the 20th week of gestation with no known causative factor and affects approximately 5% of all pregnancies (1, 2). The fundamental pathophysiological mechanisms remain unclear in approximately 50% of cases.

To date, 41 human platelet antigens (HPAs) have been described in the Immuno Polymorphism Database (IPD)^1^ (3). Several types of HPAs are involved in different clinical conditions, including venous thrombosis (4), myocardial infarction (5), post-transfusion purpura (PTP), platelet (PLT) transfusion refractoriness, alloimmune thrombocytopenia, idiopathic thrombocytopenic purpura (ITP) (6, 7), drug-induced immune thrombocytopenia (DITP), fetal/neonatal alloimmune thrombocytopenia (FNAIT) (8, 9), and susceptibility to HCV infection (10).

FNAIT is the most common cause of intracranial hemorrhage in full-term infants and can also lead to intrauterine growth retardation and miscarriage (11). FNAIT can occur during pregnancy following maternal exposure to paternal alloantigens on fetal PLTs (12).

The maternal IgG-type alloantibodies can cross the placenta and bind to the fetal PLTs. Phagocytic cells can remove these PLTs from the circulation, thus fetal thrombocytopenia occurs (13, 14). Although these results do not include the frequency of miscarriage related to the disease, FNAIT may be much more frequent than is thought, as it has been reported by some groups (15, 16). Not much data are available concerning HPA polymorphisms and recurrent abortion.

This study was conducted to evaluate the distribution pattern of HPA-1, −2, −3, −5, and − 15 [the HPA-4b alleles are rare in the Iranian population (17, 18)] in Iranian couples with a history of recurrent abortion and investigate the presence of alloantibodies in women with two or more HPA system discrepancies versus their partner.

## 2 Materials and methods

### 2.1 Study population

Informed consent was obtained from all couples. In this cross-sectional study from February 2016 to December 2017, a total of 74 couples with at least two consecutive pregnancy losses without any specified causes (according to the medical records obtained from the infertility center) were selected at the Sarem Hospital, Tehran, Iran. Ectopic, molar, and biochemical pregnancies were not included in this study.

Couples who had at least two histories of miscarriage without any known etiology were randomly selected and assigned to the present study. Their clinical situation was evaluated by infectious disease screening, e.g., tuberculosis and brucellosis history of TORCH. In addition, screening to rule out genetic disorders and clinical and para-clinical investigations regarding endocrine disease, e.g., thyroid disorders and drug toxicity, were also accomplished. If any of the mentioned diseases exist or were thought to exist, those couples were excluded from the study. The inclusion criteria for the study include the mother with no anatomical abnormality in their genitourinary system, and the father with no hypo, isospermia, or any anatomical or functional abnormal finding in their genitourinary system. After signing an informed consent, 4 mL of blood samples were collected in the EDTA tubes. Approximately at least 6 months gap was maintained between the last miscarriage and sampling.

### 2.2 HPA genotyping

Genomic DNA was extracted from the EDTA-anticoagulated whole blood samples using a QIAamp DNA blood mini kit (Qiagen, Hilden, Germany) according to the manufacturer’s specifications (extracted DNAs were stored at −20 until processing).

HPA-1, −2, −3, −5, and − 15 were genotyped using sequence-specific primers PCR (SSP-PCR), according to Bhatti et al. and Meyer et al. (19, 20). Sequence-specific primers accompanied by product values are given in Table 1. A pair of primers for the human growth hormone (HGH) was included in the PCR of each allele to serve as an internal control for the PCR. Negative control was carried out in all PCR runs. More than half of the SSP-PCR results were independently confirmed by the following confirmatory tests (RFLP, PCR sequencing, and Inotrain’s exclusive panels). These results are available as internal laboratory documentation.

**Table 1:** HPA-1, HPA-2, HPA-3, HPA-5, and HPA-15 gene primers sequence for SSP-PCR.

Gene	Primer	TM	Sequence
HPA-1	1a	62	5’ TCA CAG CGA GGT GAG GGC A 3’
	2a	63.8	5’ TCA CAG CGA GGTGAG GGC G 3’
	common	65.7	5′ GGA GGT AGA GAG TCG CCA TAG 3’
HPA-2	2a	63.8	5’ GCC CCC AGG GCT CCT GAC 3’
	2b	62.3	5’ GCC CCC AGG GCT CCT GAT 3’
	common	59.5	5’ TCA GCA TTG TCC TGC AGC CA 3’
HPA-3	3a	64.2	5′ GGG GGA GGG GCT GGG GA 3’
	3b	65.7	5′ GGG GGA GGG GCT GGG GC 3’
	common	54.6	5’ GGC CCT GGG ACT GTG AAT G3’
HPA-5	5a	53.5	5′ AGA GTC TAC CTG TTT ACT ATC AAA G 3’
	5b	54.2	5′ AGA GTC TAC CTG TTT ACT ATC AAA A 3’
	common	51.8	5’ CTC TCA TGG AAA ATG GCA GTA CA 3’
HPA-15	15a	51.7	5’ TTC AAA TTC TTG GTA AAT CCT CG 3’
	15b	51.6	5’ TTC AAA TTC TTG GTA AAT CCT CT 3’
	common	20.3	5′ ATG AAC CTT ATG ATG ACC TAT TC 3’
HGH	Forward	57.8	5’ GCC TTC CCA ACC ATT CCC TTA 3’
	Reverse	54	5’ TCA CGG ATT TCT GTT GTG TTT 3’

### 2.3 PIFT assay

PLT immunofluorescence test (PIFT) is an efficient and sensitive method in the initial screening of PLT antibody detection, but it has low specificity when non-HPA antibodies, especially Class I human leukocyte antigen (HLA) antibodies, are also present (14, 21). In the present study, the PIFT method was performed for the initial screening of PLT alloantibodies, and PRA was a candidate method to rule out non-specific positive results due to the presence of anti-HLA alloantibodies.

Whole blood was collected from six donors with ‘O’ blood group in the EDTA tubes. Then it was pooled and centrifuged at 500 *g* for 10 min. The supernatant of the PLT-rich plasma was removed, and then the buffer was washed and re-suspended in PBS/EDTA (centrifuged at 2000 *g* for 5 min and the supernatant was discarded). The washing step was repeated two times, and the final concentration was 100,000 PLTs/ml. In total, 50 μL of PLT concentrate was mixed with patient plasma (50 μL) and incubated for 30 min at 37°C (negative, positive, and isotype controls were added to each test batch).

Three consecutive washes were also performed by adding PBS/EDTA buffer (5 min at 1500 *g*). Finally, cells were incubated for 30 min in the dark with fluorescein isothiocyanate (FITC) Rabbit anti-human immunoglobulin G (DAKO F018501) at 1:30 dilution. Samples were analyzed after a final wash in a Partec PASII flow cytometer using the FlowMax software (Partec, Germany).

### 2.4 Panel reactive antibodies (PRAs)

The plasma was analyzed using a panel of mononucleated cells from 40 healthy donors. Briefly, 1 μL of isolated cells at a concentration of 3 × 10^6^ cells/ml were added to plates. The plates were incubated at 37°C for 30 min, and then 5 μL of rabbit complement was added and incubated at 37°C for 90 min. In total, 2 μL of Eosin Y solution (MERK) was added, and after 1–2 min, 10 μL of 73% formalin was added. The plate was visualized under inverted fluorescence microscopy. A PRA result of >10% was considered positive.

### 2.5 Statistical analysis

The Hardy–Weinberg equilibrium test was carried out to evaluate the distributions of the gene frequencies of HPA-1, HPA-2, HPA-3, HPA-5, and HPA-15.

Data were analyzed using SPSS version 22 software (SPSS Inc., Chicago, IL). The statistical significance of the differences between groups was calculated using Pearsonʼs χ2 test. The level of significance for all statistical tests was a *p*-value of <0.05.

## 3 Results

In total, 74 couples were included, with the mean age of women being 32 ± 7 years (ranging from 22 to 46 years), and the mean number of miscarriages being 2.5 ± 0.9. There were six Rh-negative women, and all of them had received an anti-Rh globulin injection.

Genotype frequencies for the HPA-1, HPA-2, −3, −5, and − 15 systems among studied couples were as follows: HPA-1aa: 100%, HPA-1bb: 0%, HPA-2aa: 0.6%, HPA-2ab: 96.6%, HPA-2bb: 2.8%, HPA-3aa: 24.3%, HPA-3ab: 55.5%, HPA-3bb: 20.1%, HPA-5aa: 98.6%, HPA-5bb: 1.3%, HPA-15aa: 24.3%, HPA-15ab: 46.5%, and HPA-15bb: 29.1%.

A total of 39 couples (52.7%) had partial mismatches of HPAs. Mismatches in the distribution of HPA-2, −3, −5, and − 15 among couples with a history of recurrent abortion are shown in Table 2.

**Table 2:** Allele and mismatch frequencies of HPA-1, −2, −3, −5, and − 15 in couples.

Allele frequencies (%)			*n* = 74	
	Women	men	Pairs of partial mismatches (%)	Pairs of homozygotic women with heterozygotic partner (%)
HPA-1a	100	100	0	0
HPA-1b	0	0	0	0
HPA-2a	51	51	0	1 (1.3)
HPA-2b	49	49	1 (1.3)	1 (1.3)
HPA-3a	53	52	2 (2.7)	10 (13.5)
HPA-3b	47	48	0	11 (14.8)
HPA-5a	99	99	0	0
HPA-5b	1	1	1 (1.3)	0
HPA-15a	43	51	3 (4)	6 (8)
HPA-15b	67	49	3 (4)	18 (24.3)

Among the studied couples with a history of recurrent abortion, we have noted that in HPA-1, −2, −3, and − 5, the ‘a’ allele was more prevalent than the b allele. In the HPA-1 and -5 systems, the most frequent genotype was the homozygous a/a genotype, and the homozygous b/b genotype was very rare. In HPA-2 and -15 systems, the most frequent genotype was the heterozygous a/b genotype, less frequent was the homozygous a/a genotype, and the homozygous b/b genotype was intermittent. In the HPA-3 system, the most frequent genotype was the heterozygous a/b genotype, less frequent was the homozygous b/b genotype, and the homozygous a/a genotype was intermittent.

The frequency of HPA-3a in women and men was 53 and 52%, respectively, whereas HPA-3b was 47 and 48%, respectively. The frequency of HPA-15a in women and men was 43 and 51%, respectively, whereas HPA-15b was 67 and 49%, respectively.

The common partial mismatch pairs between couples were seen as follows: 3 (4%) in both HPA-15a and HPA-15b, 2 (2.7%) in HPA-3a, and 1 (1.3%) in HPA-2b and HPA-5b. In total, 45 men (60.8%) had the heterozygote genotype of the particular HPAs, whereas their partner carried the homozygote genotype (data not shown). The frequencies of homozygote women with heterozygote partners were 24.3% in HPA-15b, 14.8% in HPA-3b, 13.5% in HPA-3a, 8% in HPA-15a, and 1.3% in both HPA-2a and HPA-2b. In 25 (33.7%) couples, mismatches were observed simultaneously for both the HPA-15 and HPA-3 systems. There was a deviation from the Hardy–Weinberg equilibrium in the HPA-2 and -5 systems (*p*: <0.0001, Table 3).

**Table 3:** Evaluation of the distribution of gene frequencies of HPA-2, −3, −5, and − 15 by the Hardy–Weinberg equilibrium test.

HPA systems	Genotypes	couples (*n* = 74)		
		EN	ON	*p*-value^*^
HPA-2	aa	34.5	1	<0.0001
	ab	72	139	
	bb	37.5	4	
HPA-3	aa	39.1	35	0.3980
	ab	71.9	80	
	bb	33.1	29	
HPA-5	aa	140	142	<0.0001
	ab	3.9	0	
	bb	0	2	
HPA-15	aa	32.6	35	0.7220
	ab	71.8	67	
	bb	39.6	42	

ON, Observed number; EN, expected number. *Pearsonʼs χ2 test.

Antibody screening was conducted for nine women who have had two or more discrepancies in HPAs with their partners to amplify the chance of alloimmunization. The PIFT and PRA results are shown in Table 4.

**Table 4:** The results of PIFT and PRA.

File no	Women’s age	Women’s blood group	Number of abortions	HPA ♀	HPA ♂	PIFT results	PRA results
4	33	O+	3	2a/2a 3a/3b 5a/5a 15b/15b	2a/2b 3a/3a 5a/5a 15a/15b	Neg	Pos
5	38	AB+	2	3a/3a 5a/5a 15b/15b	3b/3b 5a/5a 15a/15b	Neg	Neg
12	39	A-	2	3b/3b 5a/5a 15a/15a	3a/3b 5a/5a 15a/15b	Neg	Neg
14	33	A+	3	3b/3b 5a/5a 15b/15b	3a/3b 5a/5a 15a/15b	Neg	Neg
39	32	O+	2	3b/3b 5a/5a 15b/15b	3a/3b 5b/5b 15a/15a	Neg	Neg
40	32	O+	3	3a/3a 5b/5b 15b/15b	3a/3b 5a/5a 15a/15b	Neg	Neg
43	35	B+	3	3a/3a 5a/5a 15b/15b	3a/3b 5a/5a 15a/15b	Neg	Neg
55	33	A+	3	3a/3a 5a/5a 15a/15a	3b/3b 5a/5a 15b/15b	Neg	Neg
72	36	O+	3	3a/3a 5a/5a 15b/15b	3a/3b 5a/5a 15a/15b	Neg	Neg

## 4 Discussion

Recurrent abortion is a complex phenomenon that involves a wide range of possible causes. Genetic elements, such as chromosomal irregularities and parental karyotype anomalies, are frequently linked to this condition (22). Immune factors, such as autoimmune and alloimmune antibodies, can also play a significant role (23). Environmental factors, including pollution, may contribute to the risk of recurrent abortion. The interaction between genetic and immunological factors is particularly important, with immunological treatment showing promising results in preventing recurrent abortions (24).

The results of this study are valuable, as little information is available about the association between HPA polymorphisms and recurrent abortion. The present study is the first report of the gene frequencies of PLT antigens among couples with a history of recurrent miscarriage in Iran to investigate the alleles a and b antigens of HPA-1, HPA-2, HPA-3, HPA-5, and HPA-15 by the PCR-SSP method. The results showed that HPA-3 and -15 partial mismatch was common among Iranian couples with a history of recurrent abortion, and approximately half of the couples carried at least one HPA system that was absent in their partners. Primarily, partial mismatches were found on the HPA-15b, HPA-3b, HPA-3a, and HPA-15a systems. The genetic variations in HPA-3 and HPA-15 were higher than those of other HPAs, which correlated with the gene frequencies in the Iranian population.

The presence of the anti-PLT antibodies was investigated by the PIFT and PRA methods for nine women with a higher degree of HPA mismatch. The PIFT test results were negative in all tested cases, but the PRA test was positive in one case.

PLT-specific antigens have different patterns of frequency in different ethnic groups and genotyping of HPAs is important in the diagnosis of alloimmune thrombocytopenic syndromes, improving the treatment of patients with PLT auto/alloantibodies, genetic counseling for the prevention of FNAIT, etc.

Goodman C.S. et al. showed that HPA-1 polymorphism (a/b9L33P) significantly increased the prevalence of recurrent abortion compared to the control group (25). In the European white population, antibodies against HPA-1a, HPA-5b, and HPA-15b antigens were reported as the main causes of FNAIT (26). Bleeding risk may be significant throughout the severe thrombocytopenia phase, but the main risk was reported to be intracranial hemorrhage (ICH) before 20 weeks of gestation (27). In the present study, the HPA-2 heterozygous was observed in approximately 96.5% of cases, thus the possibility of alloimmunization with the HPA-2 was rare, which was consistent with the reports from Iranian blood donors by 92% heterozygosity in the HPA-2 system (17). However, Iman et al. reported an infant with FNAIT that was caused by a maternal and neonatal discrepancy in HPA-2b (28). This antigen was absent on the mother’s PLTs but it was expressed on both the father’s and baby’s PLTs, and the presence of antibody against HPA-2b in the mother’s serum was confirmed (28). The probability of FNAIT occurrence in Iran is very low due to heterozygosity in the HPA-2 system.

However, only a few cases of NAIT caused by anti-HPA-3a or anti-HPA-3b have been reported in the past (29–32). Schallmoser et al. described a term newborn with FNAIT caused by anti-HPA-3a (30). Kataoka et al. reported a case of neonatal alloimmune thrombocytopenia due to an anti-HPA-3b antibody. This infant was severely affected by ICH (32). Furthermore, Ertel et al. indicated that alloimmunization against HPA-15a and HPA-15b should be considered a cause of immune thrombocytopenia (33). In the Iranian population, Shaiegan et al. (17) revealed a distribution of 0.47.5 and 0.52.5 for HPA-15a and -15b alleles, respectively, which was not consistent with reports in Caucasian populations (34). Matsuhashi et al. reported the first case of anti-HPA-15 in Japan (35).

We also found an HPA-5b partial mismatch in this study. There is limited data on the immune response against HPA-5b, the second most frequent antigen associated with FNAIT after HPA-la. In Caucasians (36), the allelic frequencies are 0.99 for HPA-5a and 0.10 for HPA-5b, which is similar to that of the Iranian population (17). Semana et al. suggested a strong association of alloimmunization HPA-5b with a cluster of HLA (HLA DR) molecules (37). In a Caucasoid population, maternal antibodies to HPA-5b are detected in approximately 15% of serologically confirmed cases (38). Karami et al. showed that there is a relationship between rs5918 T > C polymorphism of the HPA-1 gene and recurrent abortion among the Iranian population.

HPAs are components of PLT glycoprotein (GP) complexes GPIIb/IIIa, GPIb/IX, and GPIa/IIa (3) and might be contributing to the production of autoantibodies. In addition, these antigens show a different pattern of distribution among different ethnic groups and diseases (39). Animal studies have shown that anti-GPIb alpha antibodies may increase the incidence of recurrent abortion (40). It has also been shown that trophoblasts isolated from the human placenta express αIIbβ3 integrin (41, 42). Therefore, it could be hypothesized that during pregnancy, maternal anti-αIIb and anti-β3 integrin antibodies target human villous trophoblasts, which leads to placental perfusion impairment and recurrent abortion.

As previously mentioned, there are three clinical conditions that can be brought on by PLT-specific antibodies: FNAIT, PTP, and post-transfusion refractoriness to PLTs (PTR). In short, people who lack a specific HPA allo epitope are immunized against it after receiving PLT transfusions or while carrying an antigen-positive fetus. PTR results from receiving allogeneic PLT transfusions. Class I HLA molecules typically cause these antibodies, whereas HPA molecules do so less frequently. Approximately 50–90% of patients develop HLA antibodies after receiving repeated transfusions of blood components containing leukocytes. HPA antibodies are present concurrently in 17–25% of these cases, which reduce the transfusion-associated PLT survival time (43, 44). In FNAIT, the characteristic of anti-PLT antibodies is usually anti-HLA I and, to a lesser extent, HPA antibodies, which are created during pregnancy and are based on the genetic immunity difference between the fetus and the mother (45). PTP is also closely related to immunogenicity through PLT-specific antigens. Antibodies with specificity against platelet GPs, such as GPIIb-IIIa, GPIb-IX, and GPIa-IIa, along with anti-HPA alloantibodies, are responsible for platelet destruction in PTP (46, 47).

On the other hand, it has been shown that during pregnancy, the uterine natural killer (uNK) and trophoblasts are functional partners, and the importance of uNK cells during a normal pregnancy has been determined (48). Eksteen et al. reported that anti-HPA-1a may target trophoblast function and cause trophoblast damage (49). Therefore, another hypothesis in relation to LRP is the formation of immune complexes (antibody-HPAs) on trophoblasts that trigger NK antibody-dependent cellular cytotoxicity (ADCC) on trophoblasts and as a result lead to their damage.

Deviation from the Hardy–Weinberg equilibrium regarding HPA-2 and -5 in couples was principally due to the higher number of observed HPA-2ab and -5aa alleles when compared with their expected numbers. One reason for deviating from the Hardy–Weinberg equilibrium was consanguineous marriages. This study was conducted as a pilot study, and the main limitation of this study was the small size of the study population.

It is important to note that fetal HPA genotyping is not the only consideration in the management of FNAIT and recurrent abortions. Other factors, such as maternal antibody level, maternal medical history, and fetal ultrasound evaluation, can be taken into account for comprehensive and individualized management. We then emphasize the importance of taking into consideration that decisions regarding fetal HPA genotyping and interventions to prevent recurrent abortions should be made in collaboration with a medical team specializing in maternal-fetal medicine or medical genetics, which can evaluate the specific case and provide appropriate advice based on clinical guidelines and current best practices.

In conclusion, this study revealed that HPA-3a, −3b, −15a, −15b gene polymorphisms may be involved in recurrent abortion, and further studies are essential to clarify the role of HPA polymorphisms in miscarriage, including the production of alloantibodies against HPAs.

Fetal HPA genotyping using maternal plasma cell-free DNA is recommended for alloimmunized pregnant women to determine whether their fetuses are at risk for FNAIT and whether interventions are needed to prevent recurrent abortion.

## Data availability statement

The original contributions presented in the study are included in the article/supplementary material, further inquiries can be directed to the corresponding author.

## Ethics statement

The studies involving humans were approved by this study was approved by the Ethics Committee of the High Institute for research and education in Transfusion medicine (ethical committee code number: IR.TMI.REC.1395.002). The studies were conducted in accordance with the local legislation and institutional requirements. Written informed consent for participation in this study was provided by the participants' legal guardians/next of kin.

## Author contributions

GS: Data curation, Funding acquisition, Investigation, Writing – original draft. AG: Conceptualization, Formal analysis, Project administration, Software, Supervision, Validation, Visualization, Writing – review & editing. MZ: Methodology, Supervision, Validation, Writing – original draft. MS: Formal analysis, Funding acquisition, Methodology, Resources, Writing – original draft. SS: Formal Analysis, Funding acquisition, Software, Validation, Writing – original draft. AZ: Formal analysis, Software, Writing – original draft.

## Footnotes

^1^ https://www.versiti.org/hpa
